# Structural Evidence for a Copper-Bound Carbonate Intermediate in the Peroxidase and Dismutase Activities of Superoxide Dismutase

**DOI:** 10.1371/journal.pone.0044811

**Published:** 2012-09-11

**Authors:** Richard W. Strange, Michael A. Hough, Svetlana V. Antonyuk, S. Samar Hasnain

**Affiliations:** Molecular Biophysics Group, Institute of Integrative Biology, Faculty of Health and Life Sciences, University of Liverpool, Liverpool, United Kingdom; Instituto de Biociencias - Universidade de São Paulo, Brazil

## Abstract

Copper-zinc superoxide dismutase (SOD) is of fundamental importance to our understanding of oxidative damage. Its primary function is catalysing the dismutation of superoxide to O_2_ and H_2_O_2_. SOD also reacts with H_2_O_2_, leading to the formation of a strong copper-bound oxidant species that can either inactivate the enzyme or oxidise other substrates. In the presence of bicarbonate (or CO_2_) and H_2_O_2_, this peroxidase activity is enhanced and produces the carbonate radical. This freely diffusible reactive oxygen species is proposed as the agent for oxidation of large substrates that are too bulky to enter the active site. Here, we provide direct structural evidence, from a 2.15 Å resolution crystal structure, of (bi)carbonate captured at the active site of reduced SOD, consistent with the view that a bound carbonate intermediate could be formed, producing a diffusible carbonate radical upon reoxidation of copper. The bound carbonate blocks direct access of substrates to Cu(I), suggesting that an adjunct to the accepted mechanism of SOD catalysed dismutation of superoxide operates, with Cu(I) oxidation by superoxide being driven via a proton-coupled electron transfer mechanism involving the bound carbonate rather than the solvent. Carbonate is captured in a different site when SOD is oxidised, being located in the active site channel adjacent to the catalytically important Arg143. This is the probable route of diffusion from the active site following reoxidation of the copper. In this position, the carbonate is poised for re-entry into the active site and binding to the reduced copper.

## Introduction

Cytosolic copper-zinc superoxide dismutase (SOD) catalyses the dismutation of the superoxide radical to oxygen and hydrogen peroxide via redox cycling of the copper atom, playing a vital role in the cellular defenses against oxidative stress [1]. The enzyme is a 32 kD homodimer with each subunit forming an eight-stranded Greek key β-barrel containing a disulphide bond, a solvent exposed catalytic copper atom and a buried zinc atom that is important for structural integrity [2]. Copper and zinc atoms are linked by a bridging histidine residue, which is detached from the copper during catalysis and reduction to Cu(I). A positively charged channel and ‘electrostatic loop’ guide superoxide and other small anionic substrates to the active site copper atom. A positively charged arginine residue (Arg143) in this channel is ideally positioned for electrostatic anchoring of the superoxide during catalysis [3] and has also been identified as a binding site for other anions [4], including phosphate [5], formate [6] and, when the copper atom is reduced, azide [7]. Oxidised SOD has a high affinity for small anions and cyanide, azide and thiocyanate all bind directly to Cu(II) [8]–[10] and are competitive inhibitors of the enzyme. In addition to its reaction with superoxide, SOD reacts with hydrogen peroxide (or peroxide) at the Cu site, forming a highly reactive pro-oxidant species that either inactivates the enzyme [11] through the oxidation of active site histidines and subsequent loss of copper [12]–[15], or through random or active site peptide fragmentation [16], [17]; or peroxidises exogenous substrates [18]–[20] while affording some protection against oxidative damage to the active site at physiological pH in the presence of bicarbonate [21]–[23]. In the presence of the bicarbonate-carbon dioxide pair, the oxidant has been shown to react with Trp32 on the surface of the protein [24], strong evidence of its diffusible nature. The exact structure of the bound oxidant species is unknown but is usually written as SOD-Cu(II)OH, SOD-Cu(I)O, SOD-Cu(I)-H_2_O_2_, or SOD-Cu(III) [25]. Reactivity with hydrogen peroxide is significantly enhanced in the presence of bicarbonate [17], [26]–[29], which undergoes oxidation to CO_3_^.−^ [30], [31], and it has been a matter of intense debate whether HCO_3_^.−^ [28], [29], or a HCO_4_^.−^ enzyme-bound intermediate [26], [32]–[35], or CO_2_ [25], [36] is the substrate that generates the enhanced H_2_O_2_-SOD peroxidase activity.

Carbonate or bicarbonate anions are also of general physiological interest in view of their high levels in biological fluids [37]. They are sometimes required as essential cofactors, binding in a noncovalent fashion and acting as Lewis acids or bases in enzymatic mechanisms [38] or as a structural element in the construction of metal binding sites in metalloproteins, like transferrin [39]. Carbon dioxide has several important functions in biological systems and in addition to its essential role in regulating pH it is increasingly implicated in mechanisms of *in vivo* oxidative stress, for example in the rapid non-enzymatic trapping of peroxynitrite (ONOO^−^) by CO_2_ to form the adduct ONOOCO_2_^−^. Decomposition of this adduct generates the carbonate radical anion (CO_3_^.−^), a diffusible reactive oxygen species [34]. Carbonate radicals are also produced by oxidation of bicarbonate by hydroxyl ions [37].

The suggestion that the carbonate radical is the oxidant produced by SOD peroxidase activity prompted us to investigate the nature of the interaction of (bi)carbonate with SOD using X-ray crystallography. We report here that bicarbonate binds directly at the active Cu(I) site of wild type human SOD and is also observed as a free substrate in the active site pocket when the Cu atom is oxidised. This is the first direct structural evidence for the existence of a Cu(I)HCO_3_^−^ complex at the active site of SOD, consistent with a role for a (bi)carbonate intermediate in SOD peroxidase mechanism. Additionally, the results lead us to suggest a role for bound carbonate in the reduction of superoxide to hydrogen peroxide during the dismutase activity of SOD.

## Results and Discussion

### Structural Characterisation of Carbonate Binding Sites

To obtain the carbonate adduct crystals of copper-reconstituted wild-type human SOD were soaked in potassium bicarbonate solution. The structure of an identical crystal measured prior to soaking showed the normal three coordinate Cu(I) and five coordinate Cu(II) sites typical for SOD1 (data not shown). In the bicarbonate-soaked structure, a carbonate ion was found to be present in each of the ten different copper sites in the asymmetric unit. A novel and unexpected finding was the influence of the oxidation state of the Cu centre on the mode of carbonate binding. The asymmetric unit of the 2.15 Å resolution structure contained five SOD dimers (labeled A–F, B–G, C–H, D–I, E–J) that showed differences in the structure and oxidation state of their active sites. In four of these dimers, one subunit contained Cu(II) while the other contained Cu(I), characterised in the latter by the absence of bound water, the discontinuous electron density indicating breakage of the Cu-His63 bond, and the elongation of the Cu-Zn distance [10]. In the fifth dimer (C–H), in contrast to the other four, both active sites were oxidised.

**Figure 1:**
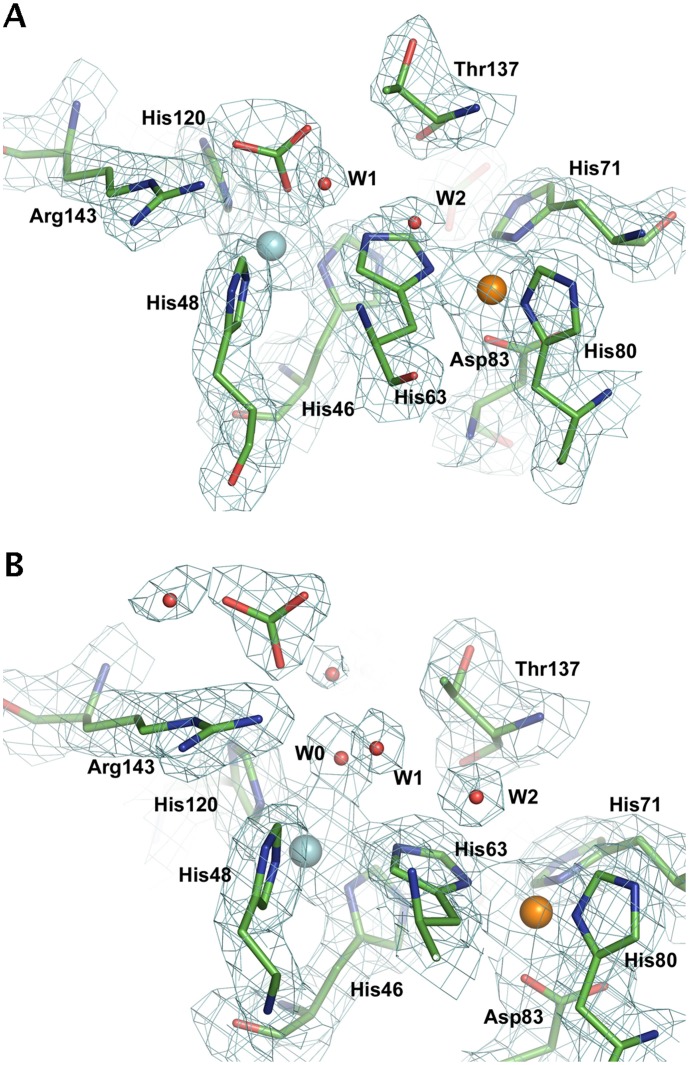
The active sites of reduced (A) and oxidised (B) SOD in the presence of carbonate. The copper and zinc ions are shown as cyan and orange spheres, respectively. In the oxidised enzyme, the carbonate anion is located ∼5 Å from the Cu(II) atom, forming H-bonds with the NE and NH2 atoms of Arg143. A well-ordered network of water molecules occupies the active site channel (also see figure 3), with W0 coordinated to the copper atom of the oxidised enzyme. In the reduced enzyme, the carbonate anion is directly coordinated to the Cu(I) atom, displacing W0. Water molecules W1 and W2 are conserved in both structures and play a significant role during superoxide binding and catalysis (see main text). The 2Fo-Fc electron density maps are shown contoured at 1.2σ.

In subunits containing Cu(II), the carbonate anion occupied a position ∼5 Å from the metal, lying between the side chains of Arg143 and Thr137, and it is held in place by interactions with the Arg143 side chain and water molecules (figure 1). The bridging His63 residue is coordinated to the Cu(II) and a water molecule provides a fifth ligand. The observation of an Arg143-bound carbonate species without direct binding to oxidised copper is consistent with earlier EPR studies at physiological pH [22], [29]. Carbonate is likely to interact with the nearby Arg143 sidechain facilitating the redox cleavage of hydrogen peroxide by anchoring it at the active copper site [29].

**Figure 2:**
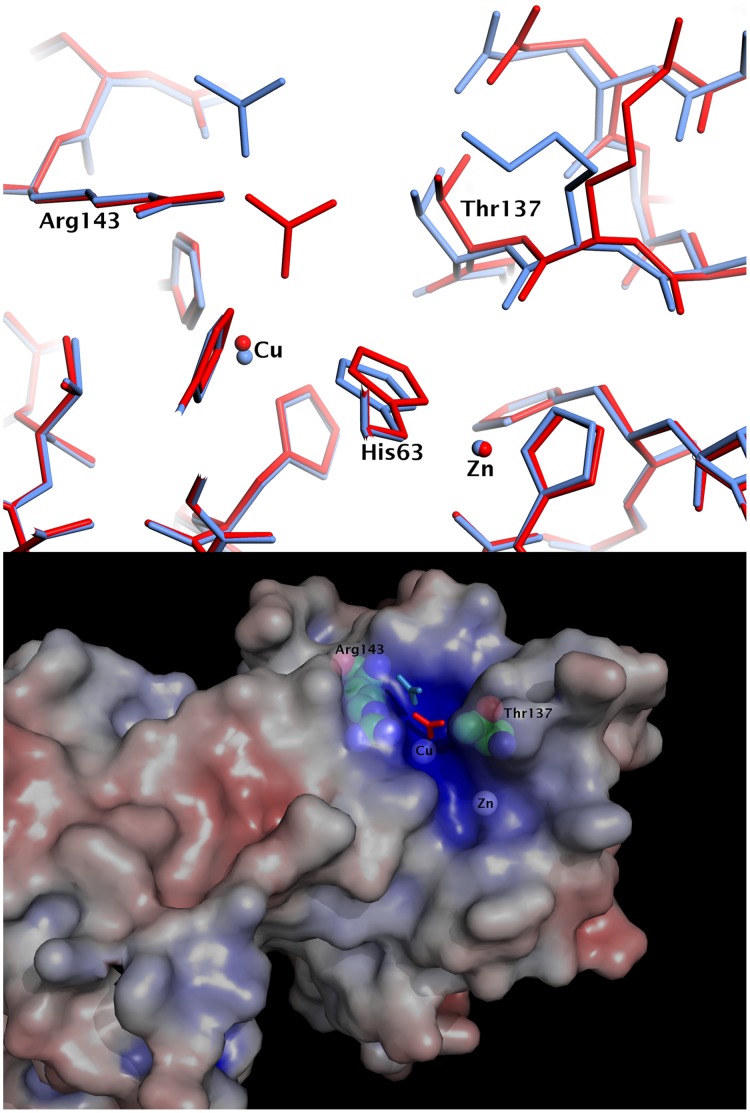
The entry and location of carbonate anions at the active site. Upper panel: Superposition of the metal binding regions of the crystal structure subunit A (red), which contains Cu(I), with subunit C (blue), which contains Cu(II). Upon reduction of the copper, the carbonate anion moves into the cavity, the copper atom moves toward carbonate and the His63 is rotated away from the copper towards carbonate. These changes are facilitated by the opening up (by ∼1 Å) of the active site cavity by a reorientation of the Thr137 main chain and neighbouring residues making up the short 3/10-helix region of the electrostatic loop. The side of the cavity formed by Arg143 and adjacent residues of the electrostatic loop are relatively unperturbed. Lower panel: The electrostatic potential of SOD plotted on the solvent-accessible surface, from +10kT/e (blue) to −10kT/e (red). The positive potential at the active site channel leading towards the copper atom is shown with both positions for the carbonate anion superimposed. The surface is displayed transparently to reveal the Arg143 and Thr137 electrostatic loop residues at the mouth of the channel, the copper atom at the base of the channel and the zinc atom.

The entry of carbonate into the copper site in subunits containing Cu(I) (figure 1) is accommodated by a conformational rearrangement of the electrostatic loop residues 132–140, resulting in an opening up of the channel entrance by ∼1 Å (figure 2). The coordination geometry of the copper has changed from trigonal planar to tetrahedral, with an oxygen atom of carbonate acting as the fourth ligand, coordinating directly to the Cu atom at ∼2.0–2.6 Å, with a Cu – O_carbonate_ – C_carbonate_ angle between 130° and 150°. The protonated His63NE2 atom is pointed towards an oxygen atom of carbonate (the separation between them is 2.5–2.9 Å for the four reduced dimers), suggesting that His63 plays a significant role in enhancing the affinity of the trigonal planar Cu(I) for this axial ligand. The carbonate ion is further anchored to the polypeptide through two hydrogen bonds between the nitrogen atoms of the side chain of the catalytically important Arg143 and second oxygen of the oxyanion. The third oxygen of carbonate is ∼2.8 Å from the CG2 atom of Thr137. The positions of hydrogen atoms cannot be determined at this resolution but it is very likely that this third oxygen of carbonate is in fact protonated, as would be expected from the pH (7.5) of the crystallisation conditions and from the absence of water or other hydrogen bond donors near to this third oxygen atom of the bicarbonate ligand.

### Significance of Two Distinct Carbonate Binding Sites in the SOD Active Site Channel

Carbonate and phosphate undoubtedly compete for the same binding sites on the enzyme [26] and peroxidative reactions of SOD carried out at physiological levels of carbonate are almost entirely suppressed in the presence of 100 mM phosphate buffer, whereas lower concentrations of phosphate show a much smaller effect [29]. Carbonate binding should predominate at the estimated physiological concentrations of intracellular carbonate, which is normally present in relatively high levels *in vivo*, typically 10–15 mM inside cells [40] and 20–30 mM in plasma in equilibrium with around 1.3 mM CO_2_ [34], versus 2.5 mM for phosphate ions [41].

A mechanism to explain the increased peroxidase activity of SOD by H_2_O_2_ in the presence of bicarbonate was originally suggested by Elam et al [26]. This proposed the formation of an enzyme-bound peroxymonocarbonate (HCO_4_^−^) oxidant that is subsequently reduced to a Cu(II)-bound carbonate radical plus an oxygen radical that oxidises endogenous or exogenous substrates. The first step in this mechanism requires the binding of HCO_3_^−^ to the reduced copper atom. The existence of this bicarbonate Cu(I)-bound species is supported by the current crystal structure (figure 1A). The positioning of bicarbonate at the Arg143 binding site in the oxidised enzyme (figure 1B) is also relevant to the mechanism of peroxidase function, as it suggests that the anion is either primed for entry into the active site upon reduction of the copper atom, or has been captured on its exit from the active site following oxidation of the copper atom. The weight of evidence is now in favour of a diffusible rather than enzyme-bound carbonate radical [35], [42] and the observation that bicarbonate binds at Arg143 rather than to Cu(II) in the crystal structure supports this view.

The SOD-Cu(I)HCO_3_^−^ binding in the crystal structure is likely to represent an *in vivo* state of the enzyme that could result when the copper site is reduced in the presence of available intracellular bicarbonate. However, Liochev and Fridovich have shown *in vitro* that CO_2_ rather than HCO_3_^−^ is the likely precursor of the carbonate radical in SOD [36]. The oxidation of CO_2_ by the pro-oxidant species formed by H_2_O_2_ at the reduced copper site may in this case still result in a carbonate-bound [25] or peroxymonocarbonate-bound [33] intermediate, which is then rapidly released following electron transfer by the copper. The current structure provides direct evidence that a (bi)carbonate Cu(I)-bound intermediate could be formed at the SOD active site. In the absence of kinetic data for bicarbonate binding to the reduced copper, the crystal structure is consistent with formation by either route (i.e. by physiological bicarbonate or by CO_2_). Whether this bicarbonate reacts in the presence of H_2_O_2_ to yield a copper-bound peroxymonocarbonate and the exact role of HCO_4_^−^ as an intermediate in the peroxidase reaction of SOD remain a matter of debate [23], [25], [32], [33], [35].

### Implications for the Dismutase Activity of SOD

The observation that carbonate is coordinated directly to Cu(I) in reduced SOD suggests a new pathway for the step in which superoxide is reduced to give hydrogen peroxide. It is well documented that superoxide, O_2_^.−^, cannot be reduced directly to peroxide, O_2_^2−^, unless it can accept a proton at the same time to form HOO^−^ or is coordinated to a metal ion that stabilises the peroxide dianion prior to its protonation [10], [43], [44]. It has previously been proposed that superoxide reacting with the reduced form of the enzyme does not coordinate directly to the Cu(I) ion but instead docks by hydrogen bonding to nearby conserved water molecules that act as the proton donors as the superoxide is reduced [10]. The current structure supports such a mechanism for this step of the catalytic cycle, whereby the bound carbonate acts as the proton donor to superoxide.

#### ‘Inner-sphere’ reduction of Cu(II) by superoxide

**Figure 3:**
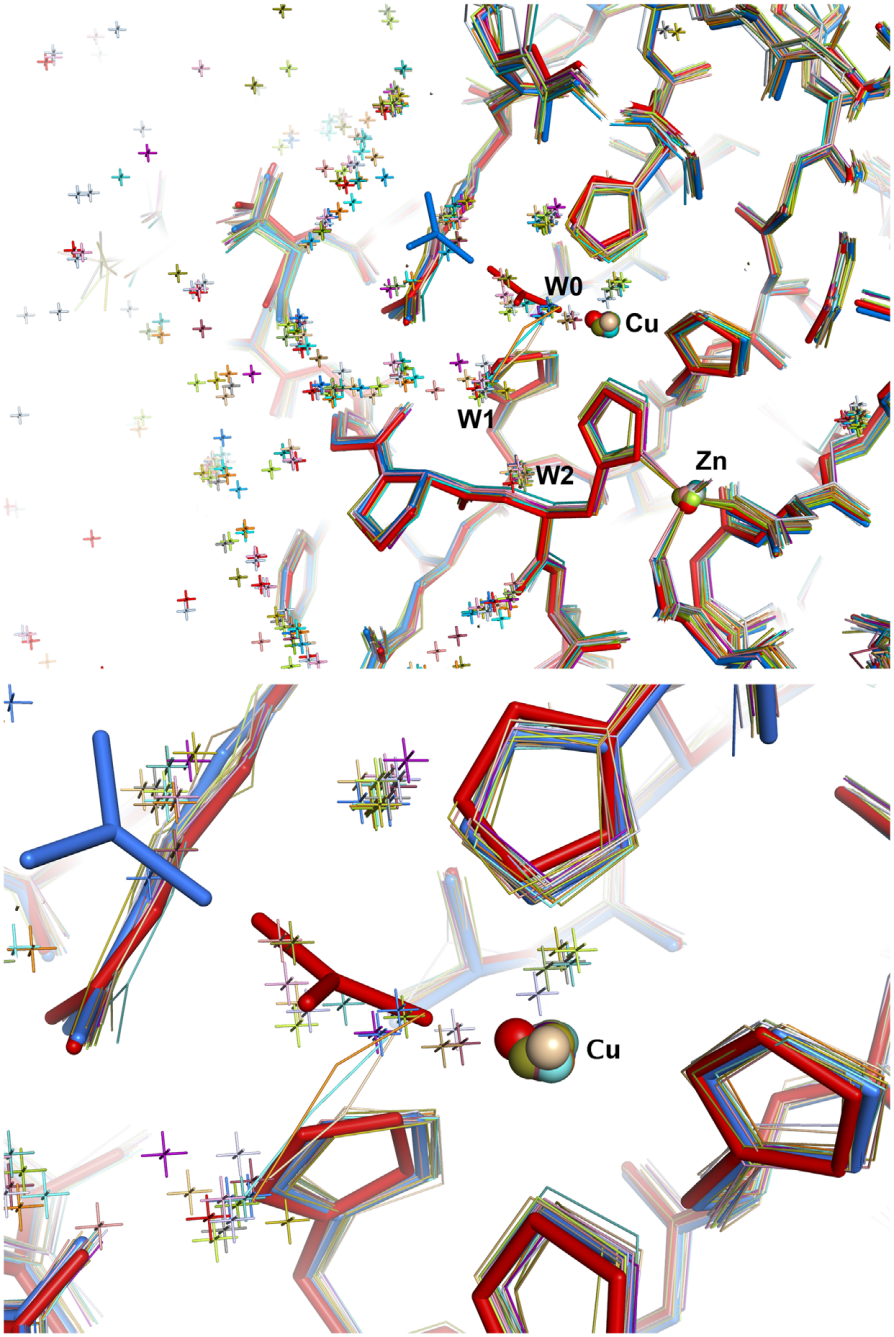
Conserved and ordered solvent molecules from the surface of the enzyme to the active site. Twenty structures of human, bovine and yeast SOD1 are superimposed. The positions of W0 (site of the first Cu(II)-bound superoxide), W1 (which we propose here is the binding site of the second ‘outer sphere’ superoxide molecule in the Cu(I) enzyme, see main text) and W2 (part of the proton channel) are indicated. The locations of azide (PDB ids: 1sxz, cyan and 1yaz, pale orange), thiocyanate (PDB id: 1sxs, orange) and carbonate anions (shown in red and blue) are also shown in close up in the lower panel: the azide and thiocyanate anions are oriented so that their terminal atoms occupy positions normally taken up by water molecules W0 and W1, while carbonate is found at position W0 in the Cu(I) enzyme (red). The superimposed structures are taken from the RSCB Protein Databank. Human SOD1∶11zv, 1n18, 1n19, 1ptz, 1pu0, 1uxm, 1hl5, 1sos. Bovine SOD1∶1sxs, 1sxz, 1e90, 1q0e, 1cb4, 1cbj. Yeast SOD1∶1yaz, 2jcw, 1yso, 1jcv, 1sdy.

**Figure 4:**
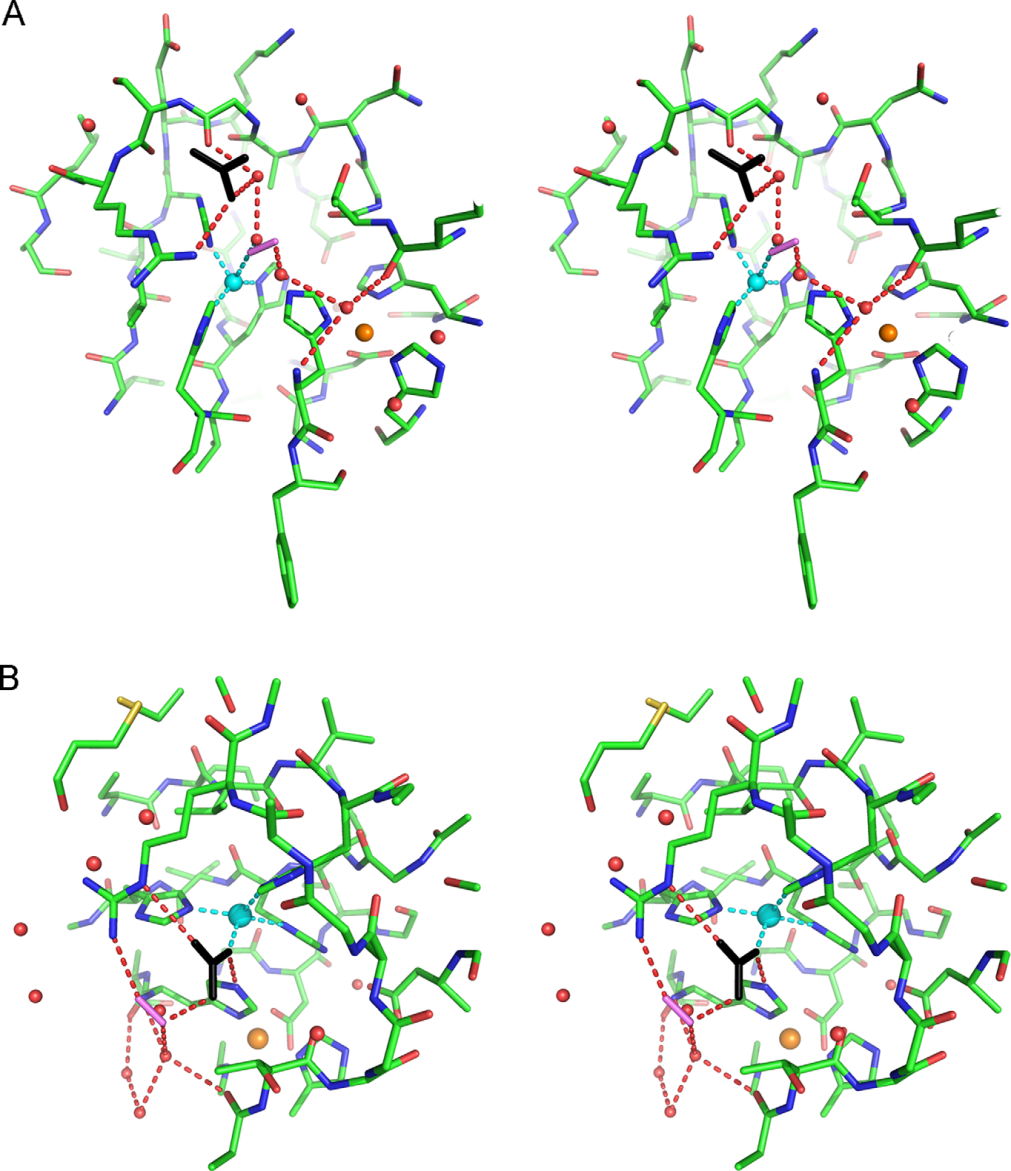
Stereo view of superoxide modeled in the SOD active site with carbonate. A 10 Å sphere about the copper atom (cyan sphere) is shown that captures the zinc (orange sphere) binding site and conserved water molecules (red spheres). The H-bonding networks around the carbonate anion (black stick) and the proposed location of the superoxide (purple stick) are indicated by dashed red lines. In the oxidised enzyme (A), the superoxide molecule is shown in position for inner-sphere electron transfer, where it is coordinated directly to Cu(II), displacing water W0 (see figure 1 for labeling scheme) and H-bonding with W1. In the reduced enzyme structure (B) carbonate is bound in place of W0 and blocks direct access of superoxide to the Cu(I) atom. The superoxide molecule is shown superimposed at the location normally occupied by conserved water W1, where it is in position to participate in outer-sphere electron transfer from the Cu(I) and simultaneous proton transfer from carbonate.

The water molecule W0 coordinated to Cu(II) is part of a well-ordered solvent network in the active site channel (figure 1). We label the two water molecules closest to W0 as W1 and W2. In the reduced subunits, the carbonate anion has displaced W0 and coordinates directly to Cu(I). Water molecules W1 and W2 remain present in both oxidised and reduced subunits and are conserved in many high-resolution crystal structures of SOD from different organisms (figure 3). When azide or thiocyanate are bound at the active site, W0 and W1 are displaced by the terminal atoms of these anions [9], [10], while W2, which is linked to the protein by hydrogen bonding to the His63 N and Lys136 O atoms, remains anchored in place. The location of W0 has been proposed to represent the binding site for superoxide in the oxidised enzyme, prior to inner sphere electron transfer [9], [10]. A superoxide molecule has been modeled in the oxidised crystal structure in this position (figure 4A) using the experimental data provided by the structures of bound anions (figure 3) as a guide. The carbonate observed in the oxidised subunit occupies a position that does not block access to inner sphere reduction of Cu(II) by superoxide.

#### ‘Outer-sphere’ reduction to hydrogen peroxide

In the next step, we propose that the Cu(I)-bound carbonate ion plays a role analogous to that of the water molecule in the standard outer-sphere mechanism, whereby the second superoxide molecule of the catalytic cycle is reduced to hydrogen peroxide. In this carbonate-based adjunct to the standard mechanism (shown schematically in figure S1), superoxide travels down the active site channel stabilized by hydrogen bonds to a chain of water molecules, oriented parallel to the Arg143 sidechain, leading from the surface of the molecule toward the carbonate ion bound to Cu(I) (figure 3). In this outer sphere scheme, the bound carbonate would block direct access of superoxide to the Cu(I) site during catalysis. Again guided by the existing structural data as summarised in figure 3, we suggest that superoxide then drops into position between the Arg143 and the carbonate ion, displacing W1 in the process. In this position (figure 4B), the superoxide would be ∼5 Å from the reduced copper atom, ∼2.7 Å from the carbonate O2 atom, ∼2.6 Å from the Arg143 and ∼2.9 Å from W2. The His63NE2 atom is ∼3.8 Å from superoxide and 2.7 Å from the O1 atom of carbonate. Simultaneously with the outer-sphere electron transfer from the Cu(I) atom, the superoxide anion receives a proton, from the O2 atom of carbonate. W2 is then able to provide a second proton to the newly formed HO_2_^−^, via the solvent chain. Meanwhile, the O1 atom of carbonate receives a proton from the NE2 atom of His63. Hydrogen peroxide is thus formed and released from the active site, the Cu(II)-His63 bond is re-formed and W0 (or another superoxide anion) replaces carbonate as a fifth Cu(II) ligand.

The carbonate anion liberated from the copper following reoxidation during dismutase activity would be able to migrate out of the active site cavity. It is interesting to speculate whether it may shuttle back and forth along the Arg143 side chain as the copper atom is sequentially reduced and oxidised during superoxide catalysis.

### Conclusion

We have demonstrated that (bi)carbonate binds in two positions, dependent on Cu oxidation state, in the active site channel of wild type human SOD, giving credence to the view that SOD may bind this anion under normal physiological conditions *in vivo*. Our data provide direct structural evidence for a suggested role for a copper-bound carbonate intermediate in the peroxidase mechanism of SOD. We speculate that carbonate may also play a role in the dismutase activity of SOD, where an addition to the accepted model is proposed for the step in which superoxide is protonated and reduced to give hydrogen peroxide.

## Materials and Methods

### Crystallisation, Data Collection, Structure Solution and Refinement

Recombinant human SOD was expressed and purified as described elsewhere [45]. Prior to crystallisation the SOD samples were Cu-reconstituted as previously described [46]. Crystals were obtained using the hanging drop vapour diffusion technique at 20°C. Droplets containing 6 mg/ml protein with 1 M ammonium sulphate, 50 mM Tris pH 8.0 and 50 mM NaCl were equilibrated over wells containing 2.0 M ammonium sulphate, 100 mM Tris pH 8.0 and 100 mM NaCl. Crystals grew in 2 weeks up to 0.2 mm in length. To obtain the bicarbonate complex crystals were soaked in 50 mM sodium bicarbonate, 2.0 M ammonium sulphate, 100 mM NaCl and 100 mM Tris pH 7.5 for up to 30 min. Immediately before data collection the SOD crystals were transferred for 10 s into a solution containing 25% glycerol.

All data were collected at the Synchrotron Radiation Source, Daresbury Laboratory, UK. Wild-type and bicarbonate-soaked Cu-reconstituted SOD datasets were initially collected using an ADSC Quantum 4 CCD detector with 0.87 Å wavelength x-rays on station 9.6. Further data were obtained on station 10.1 using a MAR225 detector. Anomalous data were collected to 2.5 Å resolution at wavelengths 1.33 Å (Cu signal) and 1.2 Å (Cu and Zn signals), from a crystal prepared in the same way, to confirm correct metallation at the Cu binding sites (figure S2). Processing of diffraction data was carried out using HKL2000 [47]. Data collection and processing statistics are shown in e I.

The structures were solved by molecular replacement using the program MOLREP [48] using the five dimers from PDB_id 1SOS as the search model. Restrained refinement and model building were carried out in REFMAC [49] and COOT [50] respectively. Multiple conformations of side-chain residues were accounted for in the last stages of refinement. Water molecules were gradually added to the models, being positioned only when well-defined positive peaks were present in both 2Fo - Fc and Fo - Fc electron density maps and when they could form hydrogen bonds with either protein atoms or other water molecules. To describe the translation, libration and screw-rotation displacement of each subunit, modeled as a pseudo-rigid body, TLS refinement was used, with each subunit treated as a single TLS group. This improved the quality of the electron density maps and the final R and R_free_-factors by 2% (table I).

**Table 1:** Data Collection and Refinement Statistics.

Resolution limits (Å)	20–2.15
(last shell)	(2.23–2.15)
No. recorded reflections	1881480
No. unique reflections	124983
Completeness (%)	94.7 (96.3)
Multiplicity	5.5 (5.4)
I/σ(I)	8.1 (2.2)
R_merge_ (%)	12.5 (68.6)
Wilson B-factor (Å^2^)	28.0
No. protein atoms	10042
No. solvent atoms	1612
No. carbonate ions	10
No. sulphate ions	5
R_cryst_ (%)	17.5
R_free_ (%)	21.5
ML based ESU (Å)	0.096
B_average_ main-chain (Å^2^)	24.3
r.m.s. deviations:	
bond length (Å)	0.010
bond angle (°)	1.334

R_merge_ = Σ|I_hkl_-<I_hkl_>|/ΣI_hkl._

R_cryst_ = Σ|F_obs_-F_calc_|/ΣF_obs._

R_free_ : R-factor using a subset of 5% of random reflections excluded from refinement.

ML ESU: Maximum likelihood estimated standard uncertainty in REFMAC [49].

Values in parentheses refer to the outermost resolution shell.

The bicarbonate SOD model was refined to an R-factor of 17.5% for all data in the resolution range 20–2.15 Å, with an R-free of 21.5%. The asymmetric unit contains ten subunits (five functional dimers) labeled A+F, B+G, C+H, D+I, E+J. The five dimers had average main chain B-factors ranging from 16.8 to 33.3 Å^2^, indicating their individual levels of disorder, related to their position in the crystal lattice. The electron density allowed the positioning of all 153 residues in each subunit with both Cu and Zn present. The final refined model contained 10042 protein atoms, 10 carbonate ions and 5 sulphate ions. There were 1612 full and partial occupancy water molecules with an average B-factor of 37.9 Å^2^. Structure validation using the MOLPROBITY server [51] gave 98.9% of protein residues in the ‘favoured’ regions of the Ramachandran plot. In subunit D, residue Ser68 was placed in the ‘generously allowed region’. The overall dihedral angle G-factor was −0.05. The estimated standard uncertainty (ESU) from maximum likelihood as implemented in REFMAC5 was 0.096 Å. Identification of carbonate ligands was confirmed using Fo-Fc omit maps for subunits A and C (figure S3) calculated after several cycles of refinement with carbonate removed from the model.

### Electrostatic Surface Calculations

Surface electrostatic calculations were performed using the using the Adaptive Poisson-Boltzmann Solver (APBS) [52] and visualised using PyMol. PDB2PQR [53] was used to prepare the SOD coordinates for input to APBS.

## Supporting Information

Figure S1 **A schematic of the proposed dismutase catalytic activity of SOD involving the bound bicarbonate anion.** The active site channel formed by the Thr137 and Arg143 residues is shown along with the relevant Cu^2+^-His63-Zn^2+^ bridge and water molecules. Other residues are omitted for clarity. Panel A: The oxidised enzyme in the resting state, with water bound to the Cu^2+^ atom and with bicarbonate H-bonded (dashed line) to the Arg143 sidechain. This corresponds to the crystal structure shown in figure 1B. Panel B: superoxide enters the active site and replaces the bound water molecule. This copper-bound superoxide has been modelled in figure 4A from the crystal structure. An electron is transferred to superoxide from the Cu^2+^ atom, which becomes Cu^1+^, and the Cu-His63 bond breaks. His63 receives a proton from the solvent. Oxygen is released from the active site. These steps constitute the ‘inner-sphere’ part of the catalytic cycle, as previously described (10). Panel C: bicarbonate enters the active site and binds directly to the reduced copper atom. The His63 is oriented towards the bicarbonate ion within H-bonding distance. This state is captured in the crystal structure shown in figure 1A. Panel D: a second superoxide molecule enters the active site, displacing a water molecule. This situation is depicted in figure 4B by a model based on the crystal structure. Superoxide accepts protons from a water molecule and the bound bicarbonate (indicated by dotted lines) and an electron is transferred to superoxide from the reduced copper atom. Hydrogen peroxide is formed and exits the active site. Bicarbonate detaches from the Cu^2+^ atom and accepts a proton from His63. The Cu^2+^-His63-Zn^2+^ bridge is re-established and a water molecule binds to the copper atom, returning to the state shown in panel A.
(TIF)

Figure S2 **Anomalous scattering difference maps for Cu-reconstituted bicarbonate-soaked human SOD1.** The maps are calculated at the 10σ level for diffraction data measured at wavelengths of 1.33 Å (blue density) and 1.2 Å (red density). Cu and Zn atoms are shown as blue and orange spheres respectively. The decrease in difference density at the position of the Cu atom is consistent with the fall-off of the anomalous signal expected from Cu in changing the x-ray wavelength and shows that only Cu atoms occupy the Cu binding site in the reconstituted enzyme.
(TIF)

Figure S3 **Fo-Fc difference maps contoured at 3**σ **with the carbonate anions omitted from the model.** Two orientations (‘flat’ and ‘end-on’) of the carbonate are shown positioned in the difference density of (A) monomer A, reduced copper and (B) monomer C, oxidised copper.
(TIF)
