# Supplementary figures and images for: Structural Evidence for a Copper-Bound Carbonate Intermediate in the Peroxidase and Dismutase Activities of Superoxide Dismutase
Source: PLoS One. 2012 Sep 11;7(9):e44811. doi: 10.1371/journal.pone.0044811 (PMC3439438; doi:10.1371/journal.pone.0044811)

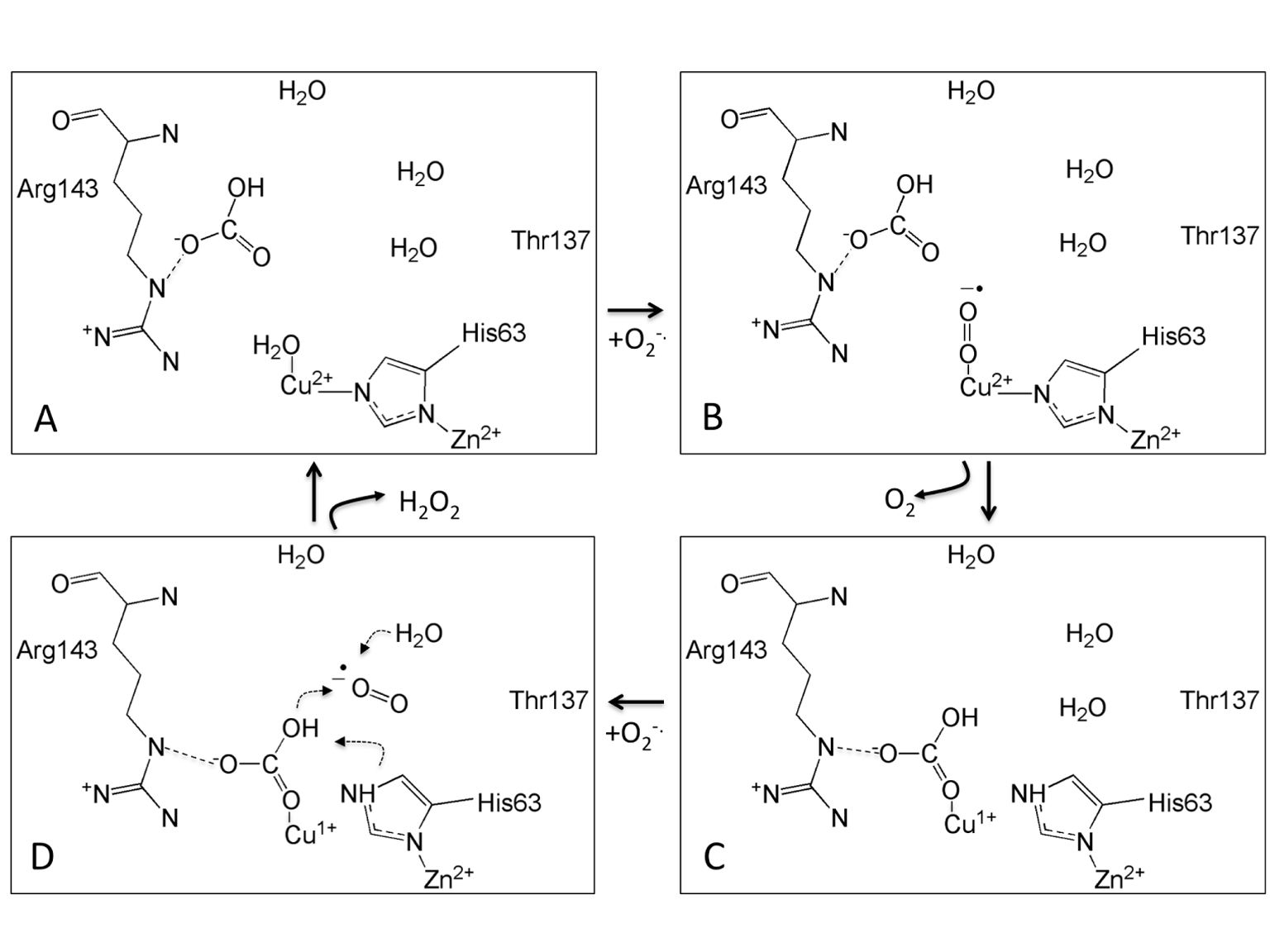

Supplement: Figure S1 — A schematic of the proposed dismutase catalytic activity of SOD involving the bound bicarbonate anion. The active site channel formed by the Thr137 and Arg143 residues is shown along with the relevant Cu2+-His63-Zn2+ bridge and water molecules. Other residues are omitted for clarity. Panel A: The oxidised enzyme in the resting state, with water bound to the Cu2+ atom and with bicarbonate H-bonded (dashed line) to the Arg143 sidechain. This corresponds to the crystal structure shown in figure 1B. Panel B: superoxide enters the active site and replaces the bound water molecule. This copper-bound superoxide has been modelled in figure 4A from the crystal structure. An electron is transferred to superoxide from the Cu2+ atom, which becomes Cu1+, and the Cu-His63 bond breaks. His63 receives a proton from the solvent. Oxygen is released from the active site. These steps constitute the ‘inner-sphere’ part of the catalytic cycle, as previously described (10). Panel C: bicarbonate enters the active site and binds directly to the reduced copper atom. The His63 is oriented towards the bicarbonate ion within H-bonding distance. This state is captured in the crystal structure shown in figure 1A. Panel D: a second superoxide molecule enters the active site, displacing a water molecule. This situation is depicted in figure 4B by a model based on the crystal structure. Superoxide accepts protons from a water molecule and the bound bicarbonate (indicated by dotted lines) and an electron is transferred to superoxide from the reduced copper atom. Hydrogen peroxide is formed and exits the active site. Bicarbonate detaches from the Cu2+ atom and accepts a proton from His63. The Cu2+-His63-Zn2+ bridge is re-established and a water molecule binds to the copper atom, returning to the state shown in panel A. (TIF) [file pone.0044811.s001.tif]

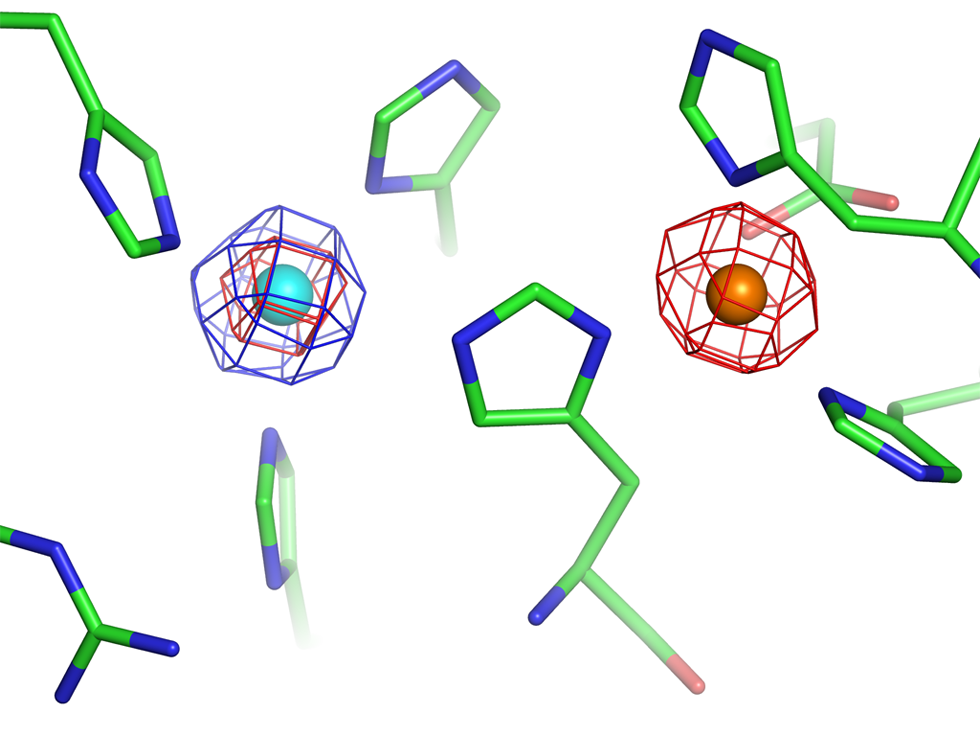

Supplement: Figure S2 — Anomalous scattering difference maps for Cu-reconstituted bicarbonate-soaked human SOD1. The maps are calculated at the 10σ level for diffraction data measured at wavelengths of 1.33 Å (blue density) and 1.2 Å (red density). Cu and Zn atoms are shown as blue and orange spheres respectively. The decrease in difference density at the position of the Cu atom is consistent with the fall-off of the anomalous signal expected from Cu in changing the x-ray wavelength and shows that only Cu atoms occupy the Cu binding site in the reconstituted enzyme. (TIF) [file pone.0044811.s002.tif]

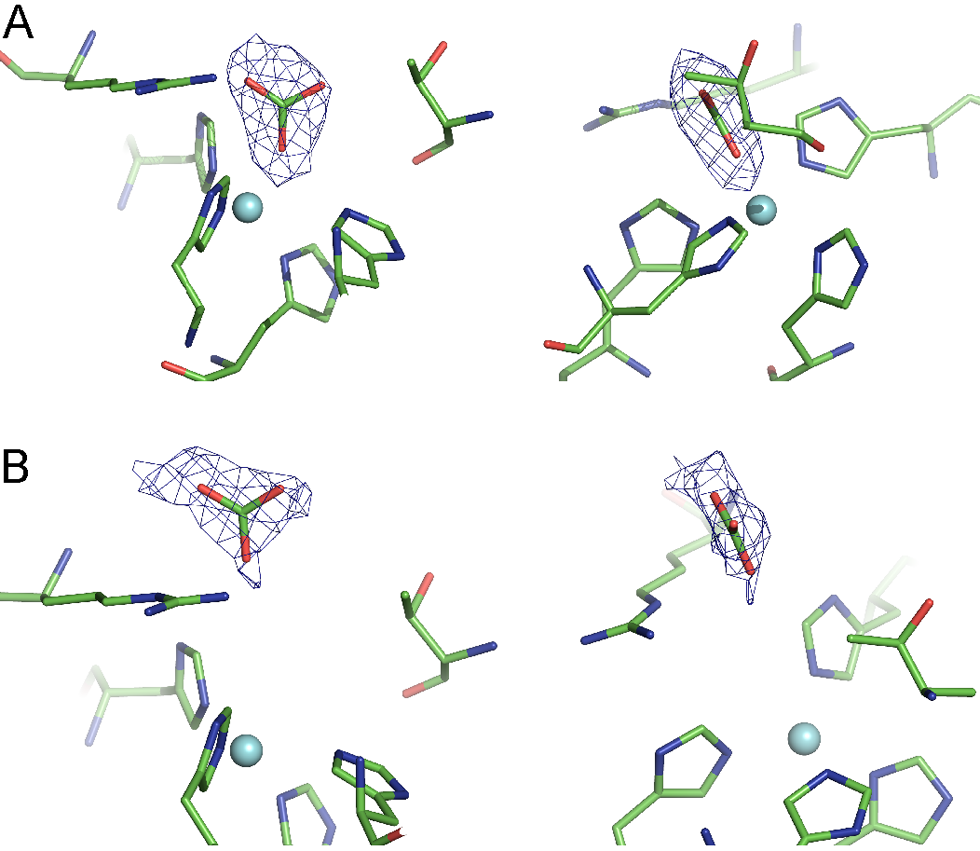

Supplement: Figure S3 — Fo-Fc difference maps contoured at 3σ with the carbonate anions omitted from the model. Two orientations (‘flat’ and ‘end-on’) of the carbonate are shown positioned in the difference density of (A) monomer A, reduced copper and (B) monomer C, oxidised copper. (TIF) [file pone.0044811.s003.tif]
